# What can be done to ease the transition to becoming a new paediatric registrar?

**DOI:** 10.1186/s12909-024-06210-7

**Published:** 2024-11-01

**Authors:** Sarah A. Long, Shouja M. A. Alam, Lynne A. Allery

**Affiliations:** 1 The Noah’s Ark Children’s Hospital for Wales, Cardiff, UK; 2 Centre for Medical Education, Cardiff University School of Medicine, Cardiff, UK

**Keywords:** Paediatrics, Medical education, Registrar, Transition, Learning needs, Interventions

## Abstract

**Background:**

The transition period of becoming a new paediatric registrar has limited study. Consequently, the learning needs of such trainees are unclear including educational interventions that may improve the process. This qualitative study examined the negative and positive experiences of transitioning paediatric trainees to identify learning needs and subsequently derive educational interventions that are perceived to ease transition.

**Methods:**

This was a qualitative study of semi-structured interviews with Wales deanery paediatric speciality trainees 3 and 4 (ST3 and ST4) undergoing transition to registrar. Participants were asked to recall one positive and one negative experience during transition using the critical incident technique (CIT). Transcribed responses were coded and thematically analysed and categorised into higher and lower order themes.

**Results:**

Six paediatric trainees were interviewed for the study. A total of eighteen codes relating to learning needs were identified and dichotomised into two higher order themes; clinical skills, and leadership and management skills, with further exploration into lower order themes. Clinical skills included child protection procedures, difficult communication with relatives, emergencies, childhood death, difficult procedures, tertiary level neonatal care, managing family anxiety and expectations, dealing with uncertainty and running clinics. Leadership and management skills involved clinical decision making by new registrars, leading ward rounds, managing workload, leading a team and supervising junior colleagues. For educational interventions, sixty-seven initial codes were recorded and combined to form thirty-two lower order themes under six higher order themes. This outlined six educational interventions perceived to ease the transition to the registrar grade including; acting up whilst a senior house officer, seniors providing feedback, seniors providing support, staff providing support, trainee familiarisation with the new registrar placement and trainees maximising SHO learning opportunities.

**Conclusions:**

This study provided a grounding upon which further research can be based, by identifying learning needs within the themes of clinical skills and leadership and management skills, as well as providing further descriptions of perceived beneficial educational interventions that ease transition to paediatric registrar. Furthermore, this study proposes evidence-based recommendations involving five key stakeholders to improve the experience of transition for future trainees. These stakeholders include; trainees, seniors, educators, nursing staff and rota coordinators.

## Background

A transition is a dynamic process where an individual is displaced from one stage to another [1]. Numerous major transitions intersperse the medical education continuum spanning from medical school up to consultant practice [2]. Postgraduate medical training in the United Kingdom (UK) traditionally involves foundation training after medical school, then specialty training before eventual consultancy. This process sees a medical school graduate become a house officer (traditionally the first year of being a newly qualified doctor), to a senior house officer (SHO) towards the end of foundation training and beginning stages of specialty training, to registrar in the later stages of speciality training, and finally to consultant who holds the ultimate responsibility for patient care. It is with each stage that the training doctor must transition to take on an increasing level of responsibility for patient care. These periods can be productive learning opportunities, but can also induce stress and even result in ‘burnout’ [3, 4]. It is therefore pertinent to understand transitions and aim to ease them.

Two well-studied transition points within medical careers include the step up from medical school to first-time doctor and the transition from registrar to consultant. The major challenges affecting new junior doctors are clinically orientated issues, while those affecting new consultants are mainly nonclinical issues [1]. However, the transition point to becoming a new registrar, in comparison, has limited study. A literature review, written in 2011, identified 73 papers exploring transitions at different stages of the medical education continuum and failed to identify research investigating the transition period to registrar [1]. Since then, few studies have further investigated this transition period with limited reference to the paediatric specialty [5–7].

The transition to registrar is a significant change in role, with increased clinical and leadership responsibilities, especially during out-of-hours shifts when the registrar becomes the most senior resident physician within most medical or surgical specialties. Inadequate preparation for this step has the potential for negative consequences and risks patient safety. Richardson et al [8] reported that 44% of obstetric trainees felt ‘*not very confident*’ or ‘*terrified’* to commence working as a registrar. Similarly, 44% of core medical trainees felt that their senior house officer (SHO) training did not prepare them to be medical registrars [9]. This contrasts 81% of paediatric trainees who reported that their SHO training prepared them for working as a registrar [10], although participants reported this in retrospect after one to two years of already being a registrar, which may have led to recall bias. This transition time is also a point at which 1 in 10 UK paediatric trainees are said to leave specialty training [11]. Those who have more positive training experiences may be better prepared and experience less stressful transition compared to their peers, and may be more likely to remain in training. Therefore, by studying and improving this transition period within paediatric training, this will improve trainee experience, attrition in paediatrics and ultimately, will have a positive effect on patient safety.

**Fig. 1:**
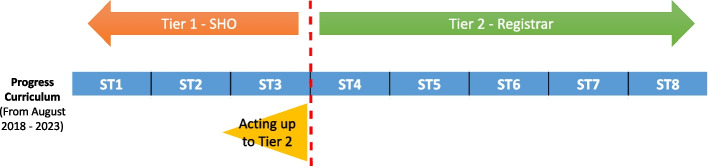
RCPCH UK paediatric training pathway at time of study. Paediatric Training within the UK following the *Progress* curriculum, as outlined by the Royal college of Paediatrics and Child Health (RCPCH). This diagram illustrates the point of training at which paediatric trainees become registrars (ST4) [12]. ST1 to ST8 refers to the ascending levels of paediatric training

Paediatric training within the UK at the time of this study, as outlined by the Royal College of Paediatrics and Child Health (RCPCH), followed the *Progress* curriculum [12]. The transition period of discussion in this study occurs between levels Speciality Training 3 (ST3) and Speciality Training 4 (ST4), moving from tier one to tier two of training respectively (Fig. 1). In the interest of clarity, tier one paediatric trainees are referred to as SHOs throughout this paper, as this is the term widely used in previous literature. Following completion of the written membership exams to the royal college (MRCPCH), one is eligible to be an ST4 trainee and registrar, and ST3 trainees can practise as registrars in ‘acting up’ opportunities (as will be discussed later in this section). It is at this time trainees must complete the final clinical exam of MRCPCH in order to progress through training.

There are a few studies available where this transition point in paediatrics is starting to be evaluated. Keene et al [13] studied 73 paediatric trainees at the end of their SHO training and reported concerns about transition, namely: leadership and decision-making, leading emergency resuscitations, working night-shifts without a senior present, difficult practical procedures, particularly neonatal intubation, and practice within community paediatrics such as safeguarding and outpatient work. Similarly, a study conducted by Menzies et al [14] found that paediatric trainees approaching the registrar level felt unprepared in the domains of leadership and management. The majority of paediatric registrars report limited or no experience with management skills in their training [15]. Additionally, Galloway and James [10] performed a national survey across the UK aiming to evaluate SHO training in paediatrics with 156 participants at the junior registrar level. The findings showed 28% of trainees wanted more time as an SHO in general paediatrics to prepare for the registrar grade while 23% wanted more sub-speciality training which appears to be an opposing request. Further evidence is needed to draw appropriate conclusions regarding these perceptions and identify learning needs.

Learning needs are specific and personal to the individual learner informed by personal practice and experience coupled with reflection [16]. Postgraduate training programmes focusing on assessing trainee learning needs are demonstrably more effective [17]. Targeting the learning needs for trainees around transition and deriving educational interventions from these needs may effectively ease the transition process. One intervention known as ‘acting up’ has already been said to improve the transition to the consultant role [18, 19]. This involves the trainee practising at a level senior to their current one [20]. It has been reported that paediatric trainees also value ‘acting up’ opportunities. However, Galloway and James’ national survey revealed that only 55% of paediatric trainees had worked in an ‘acting up’ post, but all these trainees were unanimous in describing the experience as beneficial [10]. Keene et al [13] similarly found that 62% of trainees had not experienced ‘acting up’ during their SHO training. Therefore, these data suggest that opportunities to act up are far from universal, despite being perceived as beneficial.

Furthermore, preparation courses using techniques such as simulation training have shown promise for improving the perception of transition at different levels within the medical education curriculum, especially for new doctors graduating from medical school [21–24]. However, in the UK, there are no universal courses available for paediatric trainees who are transitioning to registrars. In South Wales, there is trainee-led one day ‘transitioning to registrar’ course but this on a voluntary basis. It can be argued that courses can only be useful if they are targeted towards the learning needs of trainees. For example, Nedungadi et al [25] designed a one-day preparation course for core medical trainees stepping up to the medical registrar role using a prior pilot survey of 25 medical trainees on their views and experiences of becoming a medical registrar. The course was then tailored to the learning needs identified from this pilot survey. Post-course feedback showed an increase in self-reported confidence in undertaking the role of medical registrar from participants, with 97% stating that the course should be a mandatory part of core medical training. Specific to preparing for the role of the paediatric registrar, Menzies et al [14] and Bowman et al [26] have employed and evaluated preparation courses targeting different learning needs, including safeguarding, clinical emergencies, time management, leadership and decision making. It is unclear from both studies, partly due to only abstracts being published, as to how these learning needs were initially identified. A detailed study of the learning needs of paediatric trainees during this transition period is required to better inform such step-up courses and reform the training programme to better address these needs.

This qualitative study aimed to gain an in-depth understanding of the perceptions of paediatric trainees in South Wales, who were undergoing the transition to registrar, as to what underpins their positive and negative experiences. By doing so, we aimed to identify common learning needs during this transition period and secondly, highlight potential educational interventions that may better prepare paediatric trainees for the registrar grade and ease their experience of transition.

## Methods

This was a qualitative study using Critical Incident Technique (CIT) questioning to interview paediatric trainees about transitioning to registrar. This study was part for a Masters in Medical Education dissertation project by one author (SA). Therefore, study creation, data collection and analysis was performed solely by one author (SA) due to time, funding and resource constraints.

### Pilot Study

A pilot study was performed to refine data collection methods whilst also improving interview technique. It involved electronic open questionnaires from three individuals and semi-structured interviews of two individuals. All participants were from outside of the study population but were undergoing transition to registrar in other medical specialties. Questionnaires were initially considered as a way of improving reliability of the study through different data collection methods. However, a review of responses demonstrated poor engagement with the questionnaire lacking clarity, depth and specific examples from participants, and risked participant fatigue through repetitive questioning when combined with the interview. Conversely, it was felt the interviews alone provided richer description and greater relevance to the research question. Subsequently, it was decided that interviewing alone was better suited to researching trainee perception of transition.

### Participants

The study involved paediatric trainees currently undergoing transition to registrar in South Wales, within the Wales deanery’s School of Paediatrics. Inclusion criteria comprised ST4 paediatric trainees currently working as a registrar, or ST3 paediatric trainees working as a registrar in ‘acting up’ posts. The ST3 trainees were acting up on either an ad-hoc basis due to staffing issues or alternatively, on an elective pre-planned ‘acting up’ post. The ST4 trainees who were included had not undertaken an ‘acting up’ post whilst they were ST3 trainees. Exclusion criteria comprised: ST3 trainees that had not experienced acting up to registrar, trainees working at ST5 or above, trainees working below ST3, and those unable to attend the interview date due to prior commitments. More senior trainees, ST5 and above, were not included as it was felt that they had already spent time working as registrars to the fullest capacity and therefore, may demonstrate potential recall bias for issues that have long surpassed. ST3 trainees who had not experienced ‘acting up’ and trainees working below ST3 were not included because they had not experienced working as a new registrar yet.

### Sampling

This study acknowledges its narrow sample size and limit to data saturation. However, it can be argued that this study reaches data sufficiency rather than data saturation through the in-depth interviews with rich narrative. This is discussed in more detail within the discussion.

### Recruitment

Paediatric trainees were invited to participate in the study through advertisement at a local research conference and an email distributed by the Wales deanery’s School of Paediatrics administration team upon request by one author (SA). Recruitment was then followed by personal invitation via social media networks by one author (SA). Participation was voluntary, following distribution of a participant information sheet, and written consent obtained from each participant prior to interviews. Ethics approval was granted by the Cardiff University Ethics Committee, and ‘gatekeeper’ permission to approach paediatric trainees was provided by the head of the School of Paediatrics, within Health Education and Improvement Wales (HEIW).

### Interviews

Critical incident technique (CIT) style of questioning was adopted for the purpose of this study as the technique has been widely proven to be an effective investigative tool when interviewing healthcare professionals for research [27]. It converts rich narrative about critical incidents into thick data for thematic analysis [28], facilitating theory generation for an under-researched study population. It can involve direct observation of critical incidents. However, this was felt not to be logistically feasible in this study as these events could occur at different times throughout the day and night, and with different hospital placements and numerous participants, this would have required prolonged labour intensive observation that could not be provided due to time and resource constraints. Furthermore, authors felt that an observer present during busy clinical shifts, especially during stressful critical incidents, such as the resuscitation and death of a child, would not be appropriate.

Critical incidents to a participant are atypical events highlighted in their memory. In this study, participants were asked to recall a negative critical incident during their transition to the registrar role to capture concerns, or rather what their learning needs were at this point. Likewise, participants were also asked to describe a positive critical incident during transition and what they believed made it so. Each experience was then explored to determine the underlying educationally relevant issues by following a semi-structured interview guide of pre-determined, open-ended questions. The interviews were recorded on a mobile device and stored electronically. The audio recordings were then transcribed, anonymised and kept confidential. The interviews were held in private over the telephone for approximately forty minutes. The same interviewer (SA) conducted all the interviews.

### Analysis

The transcripts were analysed using thematic analysis in an iterative process by a single researcher (SA). A six-phase approach was adopted for thematic analysis as proposed by Braun and Clarke [29], including familiarisation with the data, generating codes then themes, then reviewing themes before categorisation including the separation of sub-themes, and finally presenting the data analysis. Familiarity with the interview data was gained through immersion. Verbatim transcript data was analysed with constant comparison between existing and emerging data, generating initial unrestricted labelling of similar data through an open coding technique. These codes were then reviewed and combined to form themes and sub-themes (higher and lower order themes respectively). Once the themes were produced, they were checked in relation to the combined codes to ensure that the relevant transcripts were fully characterised by the theme. Themes were then named and categorised with respect to learning needs and educational interventions.

## Results

**Fig. 2:**
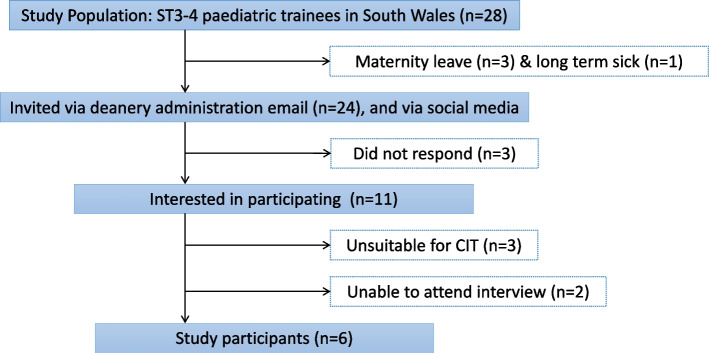
Diagram for participant recruitment and enrolment in the study

Of twenty-eight ST3 & ST4 trainees in the study population, eleven individuals volunteered to participate. Five of these participants were excluded because they were either not able to attend the interview (maternity leave, long term sick leave, prior commitments) or had not spent time 'acting up' as a registrar as an ST3 trainee (Fig. 2). The final number of study participants was therefore six trainees (Table 1).

**Table 1:** Comparison of demographics between the study population and the study sample

	**Study Sample**	**Study Population**
Total Number of Participants	6	28
ST3	2	17
ST4	4	11
Average Age (years)	31.2	31.8

A table demonstrating the similarities and differences between the study sample and the study population of paediatric trainees in the Wales deanery

This study addresses the research question by firstly, identifying the learning needs of paediatric trainees, and secondly, by identifying educational interventions to improve training and therefore, ease the experience of transition.

### Identifying learning needs

Regarding learning needs, eighteen codes were organised to form fourteen lower order themes under two higher order themes; ‘clinical skills’, and ‘leadership and management skills’ (Table 2).

**Table 2:** Identified learning needs for transitioning paediatric registrars

Higher Order Theme	Lower Order Theme
Clinical Skills	Child Protection Assessments
	Difficult Communication Scenarios with Relatives
	Emergencies
	Childhood Death
	Difficult Procedures
	Tertiary Level Neonatology
	Managing Family Anxiety and Expectations
	Dealing with Uncertainty
	Running Clinics
Leadership and Management Skills	Clinical Decision Making
	Leading Ward Rounds
	Managing Workload
	Leading a Team
	Supervising Juniors

This diagram summarises the identified higher order themes and lower order themes surrounding the learning needs of transitioning paediatric trainees within the study sample

#### Clinical Skills

The higher order theme of ‘clinical skills’ was categorised into nine subthemes: ‘child protection assessments’, ‘difficult communication scenarios with relatives’, ‘emergencies’, ‘childhood death’, ‘difficult procedures’, ‘tertiary level neonatology’, ‘managing family anxiety and expectations’, ‘dealing with uncertainty’, and ‘running clinics’.

Firstly, dealing with a ‘child protection assessment’ independently as a registrar and completing the medical examination and report writing, was considered a significant learning need for participants who suggested this was due to lack of involvement with safeguarding cases during SHO training.

- *“Doing a child protection medical… As an SHO I find that you are really sheltered and watch consultants do things in front of you… I didn’t really have to do anything about it myself, which was really unhelpful when preparing me to take on the full registrar responsibilities.”* (Participant A)
- “*Some of my colleagues have never done a child protection medical, nor have they written a child protection report or even have observed one before becoming a registrar. I think that is a major unmet learning need before becoming a registrar*.” (Participant C)

Furthermore, participants also added that child protection cases present trainees with ‘difficult communication scenarios with relatives’ and opportunities for SHOs to develop these advanced communication skills prior to registrar training were rare. This ultimately affects the confidence of the newcomer registrar facing such circumstances.

- *“Having that conversation with parents about safeguarding, what it is and why we are doing it. That is the most scary bit about it.”* (Participant A)
- *“Watch the consultants break bad news to a family. These aspects of training are probably more important to prepare for the next stage …hard conversations are rare and important training opportunities.”* (Participant E)
- *“Difficulty is, you are the senior most person on the shop floor. So you will be called and will be the one tackling the difficult conversation. But because you are new, you don’t necessarily have the wealth of experience having dealt with similar scenarios before, not to the level of having a natural flow.”* (Participant E)

Participants generally felt prepared through their SHO training to manage ‘emergencies’ as a new registrar but still felt a level of anxiety. Indeed, the most negative experience of transition, as perceived by half of the study participants, was directly related to emergencies.

- *“I do feel that actually when it comes to emergencies, we are quite well prepared.”* (Participant C)
- *“Resuscitation in our line of work is relatively rare… but is quite terrifying when it comes in.”* (Participant E)
- *“She arrested on the ward one hour into my first registrar [night] shift. I think this is the worst example I can think of.”* (Participant A)

Another infrequently occurring event for SHOs to have witnessed, yet perceived as an important learning need, was dealing with ‘childhood death’. Participants acknowledged that there is a significant emotional element to having to deal with such a scenario; however, this learning need focuses more upon the practicalities of dealing with a case of a child dying.

- *“The next thing that I’m really terrified of is the case of sudden infant death and having to deal with that. Luckily, I haven’t been involved, but that does mean that the first time I have to do it will be as a registrar.”* (Participant B)
- *“The main issue is that I don’t really have a grip of what exactly needs to be done, and then when you’re trying to counsel a mother who is deeply upset, it doesn’t look really good that you don’t really know what’s going to happen next.”* (Participant B)
- *“You don’t know how you are going to cope with it… I kind of feel like you have a lot more time to deal with the aftermath thereafter.”* (Participant B)

‘Difficult procedures’ was also highlighted as a learning need for new registrars as it is expected that practical skills are advanced compared to those at the SHO level. Furthermore, it was felt that the variation between training posts may contribute to feeling deskilled and less competent than other registrar colleagues, as well as unfamiliarity with practical equipment, such as defibrillators.

- *“Performing procedures is something I was worrying about. Partly, coming from a community job, I was feeling out of practice… That fear that if it was another more experienced registrar then we may not have required I.O. (an intraosseous needle).”* (Participant C)
- *“I was more worried about using the defibrillator that I had never used before.”* (Participant A)

Participants also singled out the management of ‘tertiary level neonatology’ as a specific concern for new registrars because it contrasts with other paediatric specialties. For numerous participants, it was felt that too long a time-period had elapsed between their SHO neonatology training and being placed back in neonates as a registrar.

- *“If my first job in ST4 was neonates, well I haven’t personally done this since ST1 and you’ll feel very much out of your depth.”* (Participant C)
- *“… neonates needs are very specific. There is no other job where you are going to be fiddling with ventilator machines and monitoring electrolytes and fluids quite as specifically. The illness are different, the physiology is different and the intensity is completely different as well.”* (Participant B)

Managing a ‘family’s anxiety and dealing with their expectations’ was also considered an important learning need especially in negative critical incidents.

- *“Dealing with the anxious family who have expectations beyond what is manageable at the time.”* (Participant E)
- *“The other thing that made it worse, which is very personal to the context, is the fact that I knew the patient and the parents… It felt like for this child we absolutely had to do our best. It almost feels like because she had arrested on the ward it was almost like we had failed her.”* (Participant A)

In addition, it was also acknowledged that an important part of becoming a registrar was ‘dealing with the uncertainty’.

- *“it's about dealing with that uncertainty of whether we are being safe, and even if we take the safest option this may be detrimental for the patient.”* (Participant D)
- *“I think the key to it was being very honest to the patient. I did not have all of the information available they were after… And it was about apologising that I couldn't be more specific than that.”* (Participant E)

The participants mentioned ‘running clinics’ as an important learning need for new registrars. However, there was a difference of opinion as to how well SHO training met this learning need. Some felt that they had regular clinic time, particularly when working in community paediatrics, but others reported the inverse, highlighting the heterogeneity of SHO training experience.

- *“Firstly, clinics. I feel, especially now that I am doing general clinics, I don’t think that my SHO training prepared me that much… I think SHOs should get more experience actually running their own clinics.”* (Participant D)
- *“I felt particularly prepared because I did community paediatrics as an ST3, so had to do numerous clinics.”* (Participant C)
- *“I did get quite a lot of practice at clinic… I do also remember people telling me about doing jobs where the clinic days were taken away, but that didn’t really happen to me.”* (Participant F)

#### Leadership and Management Skills

The theme ‘leadership and management skills’ was derived from participants describing learning needs pertaining to skills with a focus on the new registrar’s role as a senior. This theme was categorised into five subthemes: ‘clinical decision making’, ‘leading ward rounds’, ‘managing workload’, ‘leading a team’ and ‘supervising juniors’. These are skills that are assumed to have developed by the time a trainee transitions.

- “*I don't know if it's an assumption for the curriculum at the moment that these goals are meant to be gained just from experience alone.*” (Participant E)

‘Clinical decision making’ was described by participants as the significant step of having to formulate management plans independent of a second opinion and as an important part of becoming the senior most member of the shift. Participants had specific concerns about decisions leading to detrimental outcomes and having to act promptly to meet demand.

- *“If a decision needed to be made it was on my shoulders, right here and now.”* (Participant A)
- *“There is also the factor of being constantly called and bleeped to make these small decisions which doesn't give you the headspace to finish with one issue before moving on to the other.”* (Participant D)
- *“I think it’s also good for our training to allow us to have experience and time making decisions for ourselves, knowing that we are going to defer asking the consultant for a little while, so that we become more confident with our own decision making and having to think for yourself.”* (Participant C)

It was also reported that SHOs were inadequately prepared to ‘lead ward rounds’ as registrars. The skills required transcend beyond the clinical review of a particular patient on the ward to considering the ward as a system and a successful ward round requiring organisation and prioritisation skills.

- *“Things like leading ward rounds and planning for the ward is something no one ever really teaches you… Obviously, we start with the sick patients, but then how do you organise things after that.”* (Participant D)
- *“As an ST2 trainee I hadn’t actually seen any patients during a ward round. You know, you’d get the notes ready and get the results. You get the obs out, you discuss with the consultant and then you scribe. That’s it essentially… I do feel as an ST3 that was a very late point to start to learn how to assess a child for a ward round for myself.”* (Participant A)

In addition, ‘managing a high workload’ and being able to effectively prioritise jobs as a registrar were significant concerns for trainees.

- *“One of my main underpinning concerns was the workload for that night and whether I would be able to cope with that on my own being pulled in many directions… being constantly called and bleeped.”* (Participant D)
- *“It was the kind of shift you needed eyes on the back of your head is kind of thing. I find that quite overwhelming… And that is my main concern, is volume more than individual cases… You can only be in one place at a time. So I guess being able to prioritise is extremely important.”* (Participant B)

Furthermore, participants highlighted that new registrars need to develop new skills to manage people, including ‘supervising junior staff’ and ‘leading a team’ of healthcare workers. Trainees reported that supervising inexperienced juniors is even more difficult for a new registrar because they are already coping with a significant adjustment, while this supervision provides an extra burden. Although SHOs do have experience supporting more junior or less experienced SHOs as they approach ST4, this was felt to be inadequate to prepare for registrar leadership responsibilities. Participants noted that there was no such targeted intervention for these skills in SHO training.

- *“It's hard to step up as a registrar when you are out of hours when the SHO is an F2 who has no interest in Paediatrics… for example, they can't do bloods or cannulas, or can’t prescribe for children because they haven't done it very much before. As a junior registrar that's really hard because you have to fill in for all of their role, as well as taking on board a brand-new role that you are only starting to come into terms with as a registrar making more senior decisions.”* (Participant E)
- *“The other thing that I had found difficult as a registrar that we weren’t exposed to as an SHO was trying to manage workforce, particularly underperforming SHOs. For example, if you have an inexperienced SHO, or they are new to paediatrics and don’t know what we need to do, or if they are not necessarily working as efficiently as you might hope, it can be quite difficult to manage that on an out-of-hours shift.”* (Participant A)
- *“I can't think of anything specifically during my training as an SHO over the last few years that was geared specifically towards me developing the leadership skills that I would need to become a registrar.”* (Participant E)

### Educational Interventions

For educational interventions, sixty-seven initial codes were recorded and combined to form thirty-two lower order themes under six higher order themes (Table 3). This outlined six educational interventions perceived to ease the transition to the registrar grade including; ‘acting up’, ‘seniors providing feedback’, ‘seniors providing support’, ‘staff providing support’, ‘trainee familiarisation with the new registrar placement’ and ‘trainees maximising SHO learning opportunities’.

**Table 3:** Identified education interventions for transitioning paediatric registrars

Higher Order Theme	Lower Order Theme
**Acting Up**	Encourage
	Stepwise
	Support
	Resuscitations
	Elective
	Core Placements
	SHO Initiative
**Seniors Providing Feedback**	Complimentary
	Avoiding Criticism
	Validation of Independent Decisions
	Private
	Timing
	Losing Feedback from Nightshifts
**Seniors Providing Support**	Approachability
	Availability
	Directly Touching Base
**Staff Providing Support**	Staff Perception of Competence
	Awareness of Trainee Transitioning
	Skill Mix
	Sharing and Mentoring
**Trainee Familiarisation with the New Registrar Placement**	Place and People
	Protocols
	Induction and Practicalities
	Acclimatising In-Hours
**Trainees Maximising SHO Learning Opportunities**	Busy Centre
	Study/Supernumerary/Off Days
	Simulated Experience
	Situated Experience
	Reflective Practice
	Maximise Opportunities on Core Placements
	Safeguarding Team
	Overcoming Workload for Learning

This diagram summarises the identified higher order themes and lower order themes surrounding the perceived beneficial educational interventions for transitioning paediatric trainees within the study sample

#### Acting Up

‘Acting up’ as a theme was derived from participants describing the educational experience of acting up as a registrar during their SHO training as being fundamentally important to their preparation for the new role. This theme is categorised into seven subthemes: ‘encourage’, ‘stepwise’, ‘support’, ‘resuscitations’, ‘elective’, ‘core placements’ and ‘SHO initiative’.

- *“I guess most of what we learn in our training is through the job, so if you are actually able to do some of the things that you’ll be doing as a registrar in your placement, at least immediately prior to taking on those responsibilities as a registrar, whilst there is more support at hand, it makes sense that those work place competencies are likely to be much more comfortable.”* (Participant B)
- *“The positive experiences that I have experienced by acting up has far outweighed the negatives by 100-fold.”* (Participant E)

The interviewees relayed the importance of changing the culture such that the training needs of senior paediatric SHOs are prioritised and opportunities to act up are ‘encouraged’. Some participants gave specific examples of successful implementation of ‘acting up’ opportunities.

- *“Our SHOs need to be encouraged more to take the lead with things… I'm not sure how to get around this except to try and change the culture and encourage SHO from ST2 level onwards to actively take the lead.”* (Participant C)
- *“Difficult conversations are the kind of thing that skipped a level and went over our heads as an SHO… I went and asked him if he wouldn't mind if I sat in with him whilst he did that case. He said not only sitting with me but why don't you run the case and I will step in and help out if it's necessary. I think that made a world of difference.”* (Participant E)

A reported barrier to providing ‘acting up’ opportunities for SHOs was an unwillingness to expose children to a junior when a senior is available.

- “*Because there were other team members much more experienced than me, it would be one of those registrars that would very naturally start leading. You’d learn a little bit by watching their style and how things work, but it would have been really good to have the opportunity to have the chance to try and lead… It actually would have been quite nice to have the culture that you try and nurture juniors to take on that role.*” (Participant A)

When discussing ‘acting up’ as a perceived effective intervention, the participants suggested that it should be done in a graduated, ‘stepwise’ fashion, preventing the experience from being overwhelming for the SHO. One interviewee likened a non-graduated approach to ‘*starting at the deep end*’ (Participant B) and suggested the benefits of a stepwise introduction of registrar shifts starting in the most supported fashion and then gradually weaning, and that this was best for patient safety. Another trainee described how more confident he was with his own competence when entering registrar night shifts after a stepwise introduction.

- *“I first acted up whilst I was an ST2. They had an excellently well thought out stepwise approach. I first of all held the registrar bleep from 9 to 5 for the first month, then long day evening shifts for the second month, then fully on the registrar rota including nights from the third month onwards.”* (Participant E)
- *“Gradually transitioning into the job sounds like the most sensible way of doing it really. Because you’re starting off doing the job when you’re well supported, in the day, later on when you’re a bit more experienced then you do long day evenings, then moving onto nights… the safest thing really. Rather than going in at the deep end, covering for a team you don’t normally work with at night”* (Participant B)

However, a lack of ‘support’ was seen to deter SHOs from benefiting from or opting into ‘acting up’ opportunities.

- *“That [an acting up job] was offered to me but I felt that that might be too stressful. I had also been speaking amongst colleagues who had done it, and it wasn’t as supportive as it could have been. It wasn’t a positive experience. That definitely put me off the stepping up role.”* (Participant F)

‘Acting up’ was also seen as particularly valuable during emergency ‘resuscitation’ of a sick child. One participant explained the disadvantage of starting a registrar job having never taken the opportunity to act up and lead a real-life paediatric resuscitation as an SHO. However, it was suggested that acting up in simulated resuscitations can yield similar benefits.

- *“As a registrar, you lead resuscitation scenarios, so having more practice at leading them will make you better and more confident. But within the safety net of having a senior colleague around at the same time.”* (Participant B)
- *“This is an excellent opportunity as an SHO to safely take the lead in resuscitation scenarios.”* (Participant C)

Participants described ‘acting up’ shifts being offered to them as a means to fill rota gaps and preserve service provision rather than an ‘elective’ option. One participant reportedly felt almost coerced into these shifts, with the effect again of feeling like service provision had been prioritised over their training.

- *“When you're ST3 there is a lot of pressure to cover registrar shifts out-of-hours because there are rota gaps, for example if the assessment unit is not staffed that evening and there's a last-minute request for you to act up. However, what would be more educationally relevant is performing as the registrar within hours when there are people around to double check and validate your decisions. Nobody really offers you these opportunities.”* (Participant C)

It was perceived as valuable for ‘acting up’ opportunities to be offered in ‘core placements’, such as general paediatrics or neonates, rather than in speciality fields which were perceived to be of less benefit in preparation for transition.

- *“We really need to start doing this with our ST2s rather than our ST3s. Most ST3s are doing speciality jobs and they are not doing general paeds, so they have almost missed the boat to act up within hours in general paeds… I think the same would be true for neonates as an ST2, because both neonates and general paeds are amongst the core jobs that you do as a registrar.”* (Participant C)

Participants identified that it is essential for SHOs to demonstrate enthusiasm and ‘initiative’ to take on ‘acting up’ opportunities and work above their current level. Others commented that such initiative would directly translate into being better prepared for their first registrar job.

- *“The more engaged you have been with things that were probably a little above your level, or getting involved with some things that you probably didn’t strictly need to do as the SHO but took the initiative to join the registrar for the more rare things, or even for the more manageable of complex issues even tackling it yourself, puts you in good stead for when being the actual registrar for the first time, and make that transition easier.”* (Participant E)

#### Seniors Providing Feedback

‘Seniors providing feedback’ as a theme was derived from participants describing the importance and impact of feedback delivered to them during their transition and early in their work as a registrar. The six subthemes within this theme included; ‘complimentary’, ‘avoiding criticism’, ‘validation of independent decisions’, ‘private’, ‘timing’ and ‘losing feedback from night shifts’.

Participants reported a level of vulnerability with the step-up to the registrar role and how efficacious early ‘complimentary’ feedback can be in improving the situation for new registrars.

- *“When the consultant said, ‘well done, you did a good job’, it made me feel like ‘wow, yes, I can do this’… I think it would be good if all handovers were like that and that consultants were mindful that we are a bit fragile especially in the first few weeks to give us that extra effort to give feedback and affirm that we are doing well… The next time I came in for my night shift I definitely felt 100% more prepared, more confident knowing that I can do the job well to the satisfaction of my bosses.”* (Participant C)

Participants explained that new registrars are worried about being criticised. The aforementioned fragility means that ‘criticism’ can have an exaggerated negative effect on the new registrar, especially when the criticism is seen to be unfair or failing to acknowledge the effort made.

- *“I think a big part of it is deep down being worried that you will be criticised in the morning for what you did… It’s usually quite small things. Like, oh I wouldn’t have done a chest X-Ray or started antibiotics. Sometimes if its framed as teaching, like a learning point, I don’t see that as criticism.”* (Participant D)
- *“I was quite upset actually, I cried which is something that I don’t do. I think that really got to me. I think I worked quite hard and I really tried my best. So when someone says to you that that was wrong, and she basically said that I was responsible for the child going to High Dependency Unit (HDU), which I still disagree with. That was tough. And that does contribute to my fear of being a registrar.”* (Participant B)

The interviewees suggested methods of delivering feedback to make it more constructive and less likely to be perceived as criticism, such as having a structured approach involving the trainee themselves to highlight potential deficiencies and provide suggestions for improvement, and sandwiching corrective feedback amongst praise for good practice.

- *“I think if we did a debrief at the end, or decided to have a work based assessment and discussion around the event, and just go through the scenario and suggest that perhaps we need to reflect on this aspect. Something a bit more constructive and structured, rather than walking in and kind of asking 'oh, what have you been doing?'”* (Participant F)

Participants described a sense of closure that is gained by new registrars from the opportunity to have their ‘independent decisions validated’ by a senior.

- *“I would have gone home thinking was that the right decision? Did I do this OK? I would have gone through everything in my head. But having that positive feedback allowed me to put closure on it.”* (Participant C)

Participants stressed the importance of having corrective feedback delivered ‘privately’ and avoiding feedback at handovers. It was also commented on how the presence of SHOs and other consultants at the time of corrective feedback can lead to undermining.

- *“As a registrar, you feel more sensitive because it is upon you, and you are in a group of people, including the SHOs that you will be working with. You don’t really want to look stupid in front of them… and I suppose with the consultants as well, I feel a bit more that you would want them to think that you are capable and making good decisions.”* (Participant D)

The importance of choosing the right ‘time’ to deliver feedback was also highlighted by participants. It was advised against giving corrective feedback at the end of a long shift as well as waiting for new registrars to grow more comfortable in their posts before delivering more corrective feedback.

- *“Further into the post I do feel like I really would like some of more negative feedback. I do want to know what I could do differently and how I can improve. But I do think that you need to be comfortable with the team to be able to accept such feedback.”* (Participant C)

Participants explained how registrars often miss out on ‘feedback on their work during nightshifts’. The information that is assimilated during the day should be somehow relayed to the night registrar in order to achieve learning and practice development.

- *“I was making decisions at night but not really getting much feedback.”* (Participant D)
- *“It’s just that often there is a discussion around management choices, and through the working day there is an outcome about whether to change management, or not, or what would have been better to do in future. And if you are only coming in for out-of-hour shifts only, you may not get to experience that feedback and complete that cycle for your own learning. Some of this does happen at handover though, but often happens through the day.”* (Participant D)

#### Seniors Providing Support

The theme ‘seniors providing support’ was derived from participants describing the beneficial or detrimental effects of how a senior interacts and engages with a new registrar upon their perception of the transition process. This was divided into three subthemes; ‘approachability’, ‘availability’ and ‘directly touching base’.

The participants highlighted clear barriers to being able to approach a consultant for help. Trainees felt that a culture change is required amongst the consultant body to improve ‘approachability’ across the board.

- *“I did feel a little ashamed because culturally it’s not really the done thing to ask the consultant to come in unless it’s something absolutely dire happening. So I did feel a little bit embarrassed about that.” *(Participant B)

Having senior support ‘available’, either consultants or senior registrars, was reported as valuable for new registrars. However, some seemed to appreciate the lack of consultant presence to promote independent decision making, while others preferred the comfort of consultant proximity onsite.

- *“If you are given something which you are umming and ahing about, then the consultant presence actually becomes a crutch, preventing you from challenging yourself and making your own decisions, because they will be relied upon much more in these circumstances.”* (Participant E)
- *“Just knowing that you have that back up with the consultant a few minutes away in their office just put your mind at ease knowing that you're not going to have to wait for half an hour before the consultant can arrive or whether they can be contacted at all.”* (Participant C)

Additionally, the consultant ‘directly touching base’ with the new registrars prior to and during the shift was greatly appreciated by the interviewees.

- *“It just would be nice for the consultant to have talked directly to me before the night shift, on my first night as a registrar, just to set the scene and make it clear who has been informed, what I can do if I need help and when to call him. That really would have made the night a lot easier.”* (Participant D)
- *“I do remember as a new registrar in [a previous hospital], one consultant in particular who I liked doing my nights with him. He would always phone at midnight to ask how are things and be able to sound off a few small issues. He would usually as well phone in the morning at around 7am, so just being able to sound off a few questions before hand over… It would be nice if everyone did it. Perhaps not throughout your training… It would help at and around the transition period.”* (Participant D)

#### Staff Providing Support

The theme ‘staff providing support’ was derived from participants describing the beneficial or detrimental effects of how other staff members interact and engage with a new registrar upon their perception of the transition process. Staff support was divided into four subthemes; ‘staff perception of competence’, ‘awareness of trainee transitioning’, ‘skill mix’, ‘sharing and mentoring’.

The participants cared about the way ‘staff perceive’ them. A trainee reported how simple nursing staff input, such as calling the new registrar an SHO, or ‘second-guessing’ their plan can be perceived as undermining and needs to be avoided. Furthermore, other participants reported that nursing staff who compared new registrars with senior registrars have greater expectations. It was reported that if staff members ‘knew who the new ST4 registrars’ were, this would help them understand and facilitate the provision of extra support.

- *“Because nurses will compare you with ST8 trainees… The expectations would be of course quite different and so there should be more leeway and less pressure.”* (Participant C)
- *“Just an awareness especially when its change overtime with new names on the rota just being aware who is new to the role of ST4 who is transitioning in the next few weeks and who do we need to keep a closer eye on and give more support making sure that they’re OK.”* (Participant C)

The interviewees suggested that rota coordinators should ‘*couple new ST4 or ‘acting up’ registrars with more experienced paediatric trainee SHOs*’, allowing the new registrars to adjust to their new role before adding the potential burden of an inexperienced SHO. Furthermore, particularly for ‘acting up’ registrars, it was identified as important that other registrars on service be senior registrar trainees and in a position to support if needed.

- *“I’m sure there is a way to structure the first few months that means more junior registrars are paired with SHOs that can do more, meaning that the registrar is freed up more to focus on registrar responsibilities.”* (Participant A)
- *“They were told that they would only ever be on a nightshift with an experienced ST2 or ST3 paediatric training as their junior, but they turned up and had realised that swaps had been made and would be paired with a foundation doctor. And that’s not particularly ideal.”* (Participant F)

The participants highlighted the value of new registrars discussing and ‘sharing experiences’ with peers and looking to more senior registrars for ‘mentorship’. Such conversations demystified the mantle of being a registrar, and helped the newcomers adjust.

- *“It’s also reassuring knowing that other registrars also feel quite scared when they are called to resuscitations – that it’s a normal feeling—that’s quite reassuring. So perhaps some formal mentoring discussions with senior colleagues who have been through that transition previously.”* (Participant C)
- *“Having another registrar around meant that I could speak to them more on a peer-to-peer level. So having those conversations means that you can tell yourself that you don’t need to know everything. That you are very much still human and can make mistakes. You don’t need to be perfect to be a registrar and in fact nobody is.”* (Participant A)

#### Trainee Familiarisation with the New Registrar Placement

The theme ‘trainee familiarisation with the new registrar placement’ was derived through participant descriptions of how the transition to being a new registrar was eased through getting used to the placement. Four lower order themes were identified, including ‘place and people’, ‘protocols’, ‘induction and practicalities’, and ‘acclimatising in-hours’.

Participants valued having a familiarity with the ‘place and people’ where they were working as new registrars, and therefore it was seen as beneficial having worked there previously.

- *“Things that really helped there was that I was very familiar with the wards, the team, the hospital. Because I was an ST2 there for 12 months, the nurses knew my name, I knew where everything was that I needed, I knew the systems and how the nurses worked. I knew how to communicate with them effectively.”* (Participant A)

Another trainee also added that consultant support may be enhanced due to the prior relationship gained.

- *“You will also probably benefit from more support from your seniors and consultants if you have already built a relationship with them as an SHO and you have gained their trust, as opposed to you if you came brand-new without any prior relationship.”* (Participant B)

Some participants suggested systems employed in other UK trusts, where trainees are systematically kept in the same placement for the end of their SHO and beginning of registrar training.

- *“I had worked in another trust in England as a paediatric doctor and they were starting a new system which I thought was quite good. Within ST3, they were gradually shifting towards registrar responsibilities in the same hospital they would be in as a new registrar in ST4”* (Participant B)

Some interviewees expressed their frustration by not knowing the ‘protocols’ during their first registrar shifts.

- *“The registrar in the Accident and Emergency Department insisted that this is a protocol for the paediatric registrar to go. I didn't really know if this was the protocol, if this was the right thing to do… Had this occurred in other hospitals where I had worked… then I would have known what was going on and would have had an idea about the policies and protocols and therefore feel little less out of control.”* (Participant C)

The interviewees highlighted numerous limitations for traditional ‘induction’ sessions at actually familiarising new staff with the new workplace. Others focused on the ‘practicalities’ that are often inadequately covered, for example on the tour of the hospital or demonstration of defibrillator equipment.

- *“The induction hadn’t really covered these things, nor did it teach us the actual practical things that you need to do such as how to bleep people or what the policies are… You know, the practical things and stuff… I expected my badge to be there but it wasn't.”* (Participant C)
- *“On the actual induction tour, someone had just showed me from the door this is the place for resuscitation. However, I wasn't given any time to really look around the room, check the drawer, see how the phones work, where to find the equipment that I need, where to find drug charts, how to make X-Ray requests and things like that.”* (Participant C)
- *“At our induction for the trust, they quickly showed us the defibrillator. So I had seen it. However, turning it on and working it is another matter.”* (Participant A)

To better familiarise oneself with practicalities, it was suggested that there should be a period of supernumerary shadowing of a peer doing the job, as well as familiarisation with the clinical computer systems.

- *“Shadowing of a colleague for the afternoon or the morning which supplements the things covered in a traditional induction… being supernumerary and attached to someone who has worked there before who can show you the ropes and the practical elements.”* (Participant C)

One participant acknowledged her appreciation for her first night shift being a month after starting as a new registrar, suggesting that a similar template should be used for other transitioning trainees to allow ‘acclimatisation in-hours’ first.

- “*Long out of hours shifts like nightshifts should not be at the very start of the job. In my scenario, this resuscitation happened about a month after starting, which meant I had a month of working in that hospital in normal daytime hours.”* (Participant A)

On the other hand, others expressed their frustration as being on call on her first day.

- *“I think being on call on the very first day is a terrible idea. With a little bit more rota planning, they could put somebody on for who wasn’t a brand-new registrar or had worked in that hospital before… I was on-call on Wednesday evening the first day and then I was off before going straight into night shifts on Friday Saturday and Sunday night… It would have been nice to be eased into the shifts.” *(Participant C)

#### Trainee Maximising SHO Learning Opportunities

This theme was derived through participant descriptions of how interventions during their SHO training, in addition to ‘acting up’, helped ease the transition to the registrar grade. Eight themes were identified, namely; ‘busy centre’, ‘study/supernumerary/off days’, ‘simulated experience’, ‘situated experience’, ‘reflective practice’, ‘maximise opportunities on core placement’, ‘safeguarding team’ and ‘overcoming workload for learning’.

Some participants described a preference to train as an SHO in a ‘busy centre’. This is in line with the Wales deanery’s shift in recent years centralising the training to major centres to ensure that trainees benefited from the maximum exposure to pathology.

- *“I didn’t feel comfortable being in a small District General Hospital (DGH) for an entire year as an SHO… so I actually asked to be moved somewhere else… in the larger place, you have a greater variety of pathology to be exposed to.*” (Participant F)

Multiple participants also highlighted the importance of utilising non-clinical time wisely towards achieving unmet learning needs to prepare for transition. For example, ‘study/supernumerary/off days’ can be incorporated into the SHO rota. Participants gave examples of capitalising on a fully staffed rota to prioritise clinic exposure as an SHO, utilising their time to take on a management role:

- *“The rotas were quite full and I did get quite a lot of practice at clinic. I also made it quite a priority because I thought this is what I’m going to have to do as a registrar.”* (Participant F)

‘Simulation experience’ was mentioned as beneficial for helping prepare SHOs for transition by all participants. Although other contexts were mentioned, such as safeguarding and child death scenarios, simulation was  mainly reported by participants in the context of paediatric resuscitation and helped them feel more prepared to face emergencies and take on a leadership role in such scenarios.

- *“I think that is the key thing to make you feel more comfortable with an actual real resuscitation scenario and taking the lead… I am a big fan of simulation and I think it's the only thing that we have, the only line of defence in keeping our skills and our confidence up about something that we face relatively rarely but is quite terrifying.”* (Participant E)
- *“More stimulation where I am placed in quite stressful situations and taking the lead. With practice of being a little bit more focused yet more situationally aware… where we are acting up above our own training grade.”* (Participant F)

However, in certain cases such as safeguarding, even simulation training was not felt to be equivalent to practising upon real patients. Participants agreed that learning by ‘performing authentic tasks on the job’ provided a better learning experience than other methods.

- *“Child protection simulation courses are also excellent, I have done them myself, but they are no substitute for doing it for real and going through the paperwork yourself. For example, remembering to check the frenulum, or taking a measuring tape with you and writing the size, shape and colour. And when you start writing it up, you think, oh maybe I should have asked the question this way.”* (Participant C)

Participants also highlighted the importance of the SHO being engaged in order to discover learning opportunities and consider where the learning gap is.

- *“But then I asked myself, what do I need to develop to prepare me for my next job as a registrar – what’s lacking… It’s all very individualised because it comes down to your prior experiences, but it’s a change of perspective that allows you to identify those key elements to focus your attention on how to best prepare for becoming a registrar.” *(Participant E)

One participant also explained how through observation and ‘reflective practice’, she was able to extract wisdom from consultants to aid her transition.

- *“You may also get to witness the consultants deliberating over clinical decisions, and weighing up options. Even by witnessing how those consultants deal with other patients can help you extrapolate that to the next patient you see… I have seen some consultants after a ward round taking some more time to help plan more, and through observing this that helps me to develop my own prioritisation and management skills.”* (Participant D)

Participants with limited general paediatric exposure in SHO training reported anxiety about taking on registrar responsibilities. It was suggested that reconfiguring on-call shifts to ensure more ‘exposure to core placements’ such as Children’s Assessment Unit (CAU) and general paediatrics would be helpful.

- “*The fact that my general paediatric experience is less than some others has contributed towards my anxiety. I did six months at [omitted], which was very useful, but then during my time in [omitted], I did very little general paediatrics, probably less than two months in total. And since then all of my on calls have been on specialty paediatrics. So I think I’m lacking the general paediatric on-call experience and CAU experience, and I do think it’s also useful to get some A&E experience because that is what we are expected to do in registrar shifts… I do think that we should be rota’d more into CAU on call shifts*.” (Participant B)

Multiple participants advocated that SHOs should spend time with the ‘safeguarding team’ to achieve their safeguarding learning needs for transition.

- “*Join the child protection rota or the consultant who is on-call for child protection, or to do a taster today in the community to make sure that they get this experience and it should be encouraged by the end of ST3 to have performed a medical, even if this is done through a taster day or a study leave day.*” (Participant C)

It was well recognised by the interviewees that ‘overcoming workload for learning’ opportunities was a priority. For example, when paediatrics gets busy, especially in the winter, important learning opportunities for senior SHOs are missed. One participant suggested whether jobs can be redistributed such that those paediatric trainees preparing for transition have a better chance to seize the potential opportunities.

- “*What can you pass onto [non-paediatric trainees] so that you can develop the skills you are going to need later on as a registrar. E.g. asking someone to do bloods or chase results whilst you get involved with the child protection case or watch the consultants break news to a family.”* (Participant E)

Furthermore, others shared rare experiences of teaching ward rounds and teaching clinics, at least for some of the patients, where the consultant supervises the junior leading the consultations and provides feedback.

- *“I have had a few clinics that were really useful where I saw the patient and then the consultant sat in the corner and watched me.”* (Participant D)

## Discussion

This study provides a deeper understanding of trainee perceptions and experiences at transition within the Wales deanery, and thus, identifies the following learning needs of these trainees: ‘clinical skills’ and ‘leadership and management skills’. This is a recognised dichotomy in the literature on transition. A literature review of 73 papers revealed that learning needs for new junior doctors graduating from medical school revolved more around clinical skills whereas the reverse was true for senior registrars transitioning to consultant posts [1]. It is perhaps not surprising then that trainees transitioning at a midpoint between these extremes have a mixture of needs from both areas, as demonstrated in this study. Subthemes under ‘leadership and management skills’ align well with themes identified by Keene et al [13] namely, leadership and decision making, leading teams during emergencies and making decisions without senior supervision at night. Bindal et al [15] and Menzies et al [14] also identified leadership and management skills as unmet learning needs for paediatric trainees. Keene et al [13] also suggested learning needs related to clinical skills, such as inadequate practical skills, the ability to run a resuscitation, safeguarding and outpatient work. These findings correlate well with this study’s identified themes of ‘difficult procedures’, ‘emergencies’ or ‘leading a team’, ‘child protection assessments’ and ‘running clinics’.

In terms of educational interventions to improve transition, ‘acting up’ has been found to be beneficial according to previous surveys of paediatric trainees [14, 26]. However, these studies are limited in that only the abstracts are published. Ultimately, although ‘acting up’ has been identified as a beneficial tool, previous studies lacked a rich description of the intervention to fully conceptualise what it entails and how it is to be implemented. This study provides further detail, breaking down the phenomenon of ‘acting up’ into seven constituent sub-themes, for example, providing elective opportunities to act up in a supported role and using a stepwise approach from in-hours to out-of-hours.

Similarly, Galloway and James [10] listed numerous identified factors, such as ‘acting up’ posts, lack of support while acting up, senior support in general, exposure to clinics, pastoral input and psychological input, as important contributors to training experience during transition, but offered no further insight into each. This study adds to these findings by presenting a more detailed understanding of the phenomena under the themes ‘acting up’, ‘senior support’, ‘running clinics’, ‘providing feedback’ and ‘sharing and mentoring’, suggesting specific practices that are perceived as positively or negatively impacting the transition process.

Findings from this study were inconsistent with previous studies. For example, although this study agreed with Galloway and James [10] who reported that one third of trainees felt that they had insufficient time in general paediatrics, Keene et al [13] reported that 93% of trainees felt competent with day-to-day work in general paediatrics regarding their transition. This may be explained by the fact that paediatric trainees will train in different hospitals in different training pathways, leading to variable exposure to general paediatrics among trainees. Furthermore, Galloway and James [10] found that trainees valued more training in primary care, mental health and public health, whereas this was not captured in our dataset. These may reflect more general learning needs rather than those specifically tailored towards transition, as was the case in this current study, but may also be reflective of potentially narrow sampling.

This study highlighted other themes not previously identified by other authors [10, 13]. The qualitative approach using exploratory semi-structured interviews permitted adequate deviation to facilitate holistic exploration of participant viewpoints on a variety of issues pertaining to their transition. As such, additional themes were generated including, the importance of ‘trainee familiarisation with the new registrar placement’.

Each of the six identified themes relating to educational interventions provides targets for further research. For example, one participant stated that they made an informed decision not to take on an ‘acting up’ job as an ST3 trainee. It is important to acknowledge the potential negative elements of ‘acting up’ that have been previously reported by trainees. Among the 51% who had worked out-of-hours on a registrar rota during their SHO training, two-thirds of these trainees felt unsupported during their out-of-hours work [10]. Further research into the negative experiences of ‘acting up’ and what deters some trainees would be insightful.

Another surprising finding was the differing views as to whether participants valued senior proximity during their transition, which is particularly relevant as regulatory body guidance is shifting towards more consultant presence; therefore, the educational impact of this upon transitioning trainees can be further examined.

It should be highlighted that this study took place prior to the introduction of RCPCH *Progress* + curriculum (updated August 2023) [30]. This updated curriculum sees tier one trainees able to act up and take on further responsibility from an earlier stage, at ST2, despite less SHO training. Further research with such trainees would build on the findings of this study to refine ways to ease transition to registrar for this cohort.

### Limitations

This study recruited only six participants and therefore may not have reached data saturation. However, Mason argues that a narrow sample size may well be appropriate for in-depth examination for a specific social process [31]. Although, the sample should be broad enough to permit adequate exploration of the phenomenon [32]. A previous qualitative study by Guest et al [33], demonstrated that out of 100% of codes generated from sixty interviews, 73% were obtained by six interviews, and 90% by twelve interviews. Duration of the interview can be used as a proxy marker for interview depth. Guest et al. did not include the duration of interview time required to achieve the 90% of codes but focussed on the number of interviews. Six in-depth interviews with thick, rich description of experiences given by this study may very well highlight more than twelve less penetrating interviews. It can therefore be argued that this study reaches data sufficiency rather data saturation [34] given that the generated themes not only confirmed what was available in the existing literature, but also highlighted previously unpublished themes.

All participants had experienced SHO training across the training centres in South Wales, but no trainees were recruited from North Wales or other parts of the UK. Additionally, further details about the participants’ educational and training histories at different training sites within South Wales were not collected. As previously discussed, this may lead to variable exposure of educational and training opportunities amongst trainees. Despite this, given that the RCPCH curriculum, General Medical Council (GMC) guidance, and processes for annual review of clinical progression are shared across all these areas, it is likely that training placements are similar. This is why the GMC ‘National Training Survey’ which is completed by trainees, is applicable for assessing and comparing all UK training units against common standards. Therefore, the identified learning needs and educational interventions from this study, although sourced in Wales, are arguably relevant to other UK deaneries. However, this may be limited to the UK as paediatric training in other countries may differ.

None of the participants had experienced neonatology during ST3 or ST4, but discussed their thoughts about having to return to neonatology after a prolonged period of not being placed in tertiary level neonatology. This is a limitation of the study, as trainees who have experienced a tertiary neonatology job at ST3 or ST4 may have highlighted additional learning needs.

It is recognised that the method of sampling laid the potential for bias. By approaching applicable trainees within the Wales deanery via social media networks, this may have excluded the views of other trainees who did not use social media networks. Furthermore, it is possible that the perspectives of an important group of trainees were missed: those who left paediatric training at the point of transition. It may have been that such trainees experienced such poor preparation for their transition that it was a factor in their attrition from the training programme.

Additionally, recall bias may have occurred when interviewing ST4 trainees who were approximately nine months from the time they initially experienced transition (to the time of the interview). Interviews also occurred over the telephone thus disallowing consideration of body language in participants’ responses. It should also be noted that participants who were unable to attend the interview were excluded from the study which could have excluded the views of trainees struggling with the increased workload of being a new registrar. As this study was part of a Masters dissertation project, authors were limited by time, funding and resource constraints, which meant the interviews and subsequent analysis was performed by one researcher. This could have led to experimenter bias.

### Recommendations and Practical Implications

Drawing on the themes generated from this study, five key stakeholders were identified when considering who may impact this transition period. These included: *trainees* themselves approaching or experiencing transition, consultants and registrars as *seniors*, *nursing staff*, *educators* such as the head of school, training programme directors, college tutors or course organisers, and finally *rota coordinators* who organise shift pattern and organisation of the work force within the department. With the findings of this study, authors suggest recommendations and propose strategies for changes in practice for each stakeholder (Table 4). These have been subcategorised according to the identified higher order themes.

**Table 4:** Recommendations for stakeholders involved in paediatric training

Themes	Recommendations	Proposed Strategies
Clinical Skills and Leadership and Management Skills	• ***Trainees*** should take responsibility to identifying their learning needs when approaching transition. • ***Seniors*** should be aware of trainees learning needs and encourage them with opportunities to work on these. • ***Educators*** could facilitate ways for trainees to develop leadership and management skills.	• ***Trainees*** could compare their competencies against this list of learning needs and use it to structure a personal development plan. • ***Seniors*** aware of these training needs can facilitate trainee involvement in such cases that present themselves, for example safeguarding or child death. • ***Educators*** could provide study days, simulation days or step-up courses for trainees approaching transition to registrar.
Acting Up	• ***Trainees*** should take the initiative to sort ‘acting up’ opportunities and discuss these with seniors in advance to increase their chances. • ***Seniors*** must encourage trainees to take the lead whenever it is safe to do so. Support the junior, facilitate the learning and stress your availability to take questions or discuss issues, especially out of normal working hours. Discuss staff shortages with the SHO and do not assume consent that they will act up as the registrar. • ***Nursing staff*** should support junior trainees who are acting up. • ***Educators*** could facilitate ‘acting up’ opportunities. • ***Rota coordinators*** should not be routinely offering ‘acting up’ shifts out of hours to simply fill rota gaps.	• ***Trainees*** could volunteer for ‘acting up’ opportunities and offer to lead a resuscitation under senior guidance. • ***Seniors*** should allow trainees to lead assessments in clinic or ward round, and try to free-up the trainee to join them in an event of educational value, such as difficult conversation with a family. Seniors should inform all consultants on-call out of hours that a junior is acting up. • ***Nursing staff*** should contact the SHO first for a senior level task if safe to do so. • ***Educators*** could offer regular ‘acting up’ opportunities in core placements of general paediatrics and neonatology. • ***Rota coordinators*** should ensure that exposure to out of hours ‘acting up’ shifts occur in a stepwise manner.
Seniors Providing Feedback	• ***Trainees*** should keep track of patients or issues they would like to receive feedback on. • ***Seniors*** should respond to trainees asking for feedback in a timely manner. Debriefs after significant events should not be delayed and should be at a convenient time for the trainee. Seniors should use complimentary feedback with validation and affirmation of the trainee’s own decisions. Avoid language that is suggesting a trainee is to blame.	• ***Trainees*** could keep a log of patients or issues from overnight that they would like feedback on, in order to follow the cases and seek feedback to complete the learning cycle. • ***Seniors*** should give feedback using a structured approach like a work-based assessment, invite trainees themselves to suggest deficiencies or practice changes, and sandwich corrective suggestions with acknowledgment of the trainee having worked hard and done their best. Giving corrective feedback should be avoided at the end of long shifts, in group settings, or early in the trainee’s new post.
Senior Providing Support	• ***Trainees*** must not feel embarrassed to contact the consultant if needed, including at night. Patient safety must be prioritised over a sense of pride or shame. • ***Seniors*** should not criticise trainees for contacting them out of hours, nor portray frustration at help being sought.	• ***Seniors*** should answer the phone (ensuring it is on) if called out of hours. Routinely touch base with new registrars directly prior to and during a shift and stress your availability for any questions or concerns.
Staff Providing Support	• ***Seniors,*** particularly senior registrars, should support trainees undergoing the transition process to registrar. • ***Nursing staff*** should be made aware of the new registrars and those acting up so that they can have appropriate understanding and give leeway, so as to not have the same expectations as with an ST8 registrar. • ***Rota coordinators*** can ensure an appropriate on-call team skill mix when registrars are new or 'acting up'.	• ***Seniors*** particularly senior registrars, can consider formally or informally mentoring transitioning juniors, discussing with them and sharing their own experiences helping to demystify the process. • ***Nursing Staff*** should avoid behaviour that can be perceived by the new registrar as undermining, for example referring to them as an SHO, or if questioning the reasoning behind the registrar’s plan. • ***Rota Coordinators*** could place new or ‘acting up’ registrars with an experienced SHO (usually a paediatric trainee opposed to a foundation or general practice trainee) on the same shift. Also, if a second registrar is on site, rota coordinators should ensure they are senior to the new/’acting up’ registrar.
Familiarisation with the New Registrar Placement	• ***Educators*** should place trainees in a familiar department for their first registrar or ‘acting up’ post. Induction should focus on trainee familiarisation of the department including equipment, protocols of the workplace and a signpost for later reference, particularly out of hours. • ***Rota coordinators*** should avoid placing new registrars on out of hour shifts early on into their rotation.	• ***Educators*** should ensure the first registrar placement is in a hospital where the trainee has previously worked. If this is not possible, can the trainee work at that hospital as an SHO grade for some weeks prior to starting as a registrar? An induction tour should allow trainees time to explore the environment and try equipment. This should be followed by a period of supernumerary shadowing of a doctor performing real tasks in order to pick up the practicalities of the new workplace. • ***Rota coordinators*** could rota new registrar into their first out of hours shift after a month of working as a new registrar in normal working hours.
Maximising SHO Learning Opportunities	• ***Trainees*** should take the initiative to actively seek out opportunities to fulfil their PDP with respect to transition during their SHO training and reflection on these experiences. • ***Rota coordinators*** should prioritise time for trainees’ professional development over service provision.	• ***Trainees*** should utilise study days or off days, or when they are supernumerary and dispensable from the ward to seek out situated experiences of actually doing tasks (such as joining the safeguarding team and performing a child protection assessment), or attend simulation training practising as a role above their training grade. Delegate lower order tasks to non-paediatric trainee SHO colleagues, like bloods and cannulas, so you can seize learning opportunities performing or witnessing higher order tasks, such as breaking bad news. • ***Rota coordinators*** should embed opportunities for study and clinic into the rota. CAU shifts should be spread between all paediatric SHO trainees including those working in different paediatric specialities.

A table outlining the proposed recommendations for five potential stakeholders involved in paediatric training, categorized into the learning needs and educational interventions identified in this study

## Conclusion

In conclusion, this study explores the views of paediatric trainees transitioning to the role of paediatric registrar, and what helps to ease this transition. Through critical incident technique questioning of paediatric trainees and subsequent thematic analysis, this study both aligns with and builds on previous research, identifying fourteen learning needs categorised under two themes: ‘clinical skills’ and ‘leadership and management skills’. Additionally, six educational interventions were identified, including ‘acting up’, ‘seniors providing feedback’, ‘seniors providing support’, ‘staff providing support’, ‘trainee familiarisation with the new registrar placement’ and ‘trainees maximising SHO learning opportunities’. Consequently, this study provides a basis for further research and proposes evidence-based recommendations to improve the experience of transition for future paediatric trainees.

## Data Availability

The datasets generated and analysed during the current study are not publicly available due to the agreed conditions at institutional ethics approval for data storage on the secure university server, but are available from the corresponding author, Sarah Long, on reasonable request.

## Abbreviations

UK: United Kingdom
SHO: Senior House Officer
RCPCH: Royal College of Paediatrics and Child Health
ST3/4: Specialty Training Level 3/4
MRCPCH: Membership of the Royal College of Paediatrics and Child Health
CIT: Critical Incident Technique
HEIW: Health Education and Improvement Wales
HDU: High Dependency Unit
CAU: Children’s Assessment Unit
DGH: District General Hospital
GMC: General Medical Council
